# Anacardium microcarpum extract and fractions protect against paraquat-induced toxicity in Drosophila melanogaster

**DOI:** 10.17179/excli2016-684

**Published:** 2017-03-20

**Authors:** Katiane Raquel Müller, Illana Kemmerich Martins, Nathane Rosa Rodrigues, Litiele Cezar da Cruz, Valter Menezes Barbosa Filho, Giulianna Echeverria Macedo, Gustavo Felipe da Silva, Jean Paul Kamdem, Irwin Rose Alencar de Menezes, Jeferson Luis Franco, Thaís Posser

**Affiliations:** 1 Oxidative Stress and Cell Signaling Research Group, Centro Interdisciplinar de Pesquisa em Biotecnologia, Universidade Federal do Pampa, Campus São Gabriel, 97300-000 São Gabriel, RS, Brazil; 2 Departamento de Química, Programa de Pós Graduação em Bioquímica Toxicológica, Universidade Federal de Santa Maria, 97105-900 Santa Maria, RS, Brazil; 3 Departamento de Ciências Biológicas, Universidade Regional do Cariri, 63100-000 Crato, CE, Brazil; 4 Departamento de Química Biológica, Universidade Regional do Cariri, 63100-000 Crato, CE, Brazil

**Keywords:** cajuí, Drosophila melanogaster, antioxidant, neuroprotection, herbicide

## Abstract

*Anacardium microcarpum* Ducke (Anacardiaceae) is a native species of Brazil used in folk medicine for the treatment of several illnesses although its antioxidant activity has been reported *in vitro*, there is no evidence of this effect in an *in vivo* model. Here, we investigated the potential protective effect of hydroalcoholic extract (AMHE), methanol (AMMF) and acetate (AMAF) fraction of *A. microcarpum* against paraquat toxicity on survivorship, locomotor performance, antioxidant enzymes activity and reactive species using *Drosophila melanogaster*. Flies were exposed to the extract or fractions (1 and 10 mg/ml) in the presence or absence of paraquat (5 mM) in sucrose solution for 72 h. In addition, total phenolic content of extract and fractions was evaluated as well as ABTS radical scavenging capacity. Our results demonstrated that AMAF presented higher content of phenols and ABTS chelating potential. Treatment of flies with the extract or fractions did not alter the survivorship, locomotor ability, and acetylcholinesterase (AchE) activity per se. Paraquat caused 85 % mortality of flies and 30 % increase in reactive species generation, which were significantly attenuated by AMHE and AMMF. AAMF increased catalase activity (from 66.77 ± 6.64 to 223.94 ± 25.92 mU/mg of protein), while AMAF increased GST activity (from 477.76 ± 92 to 770.19 ± 147.92 mU/mg of protein) and catalase activity (from 66.77 ± 6.64 to 220.54 ± 26.63 mU/mg of protein). AMHE and AMMF were more effective in protecting against paraquat toxicity. Taken together, the data indicate the potential of this plant in acting as a protective and antioxidant agent *in vivo*.

## Introduction

The genus Anacardium (family: Anacardiaceae) is composed of a relatively small number of species (about 20) native to tropical regions of the Americas. One of its species that has generated considerable interest in the past few years is *Anacardium microcarpum *Ducke. It is a native species from Brazil, found in the Northeast region where it is popularly known as “cajuí”*. *The fruits of this species differ from those of *Anacardium occidentale *L., the most representative species from this genus, by its smaller drupe and hypocarb, making it commercially less valuable (Vieira et al., 2014[35]). The infusions of barks of *A. microcarpum* have been used in Brazilian folk medicine for the treatment of a variety of illness, such as inflammation, rheumatism, tumor and infectious diseases (Barbosa Filho et al., 2014[6]).

Although the pharmacological properties of *A. occidentale* is well documented, very little is known for *A. microcarpum*. Recently, our research group demonstrated the antioxidant potential of *A. microcarpum *and absence of toxicity in a variety of *in vitro* assays system (Barbosa Filho et al., 2014[6]). Moreover, it presented synergism with commercial antibiotics, against resistant bacterial strains (Barbosa-Filho et al., 2015[7]). Additionally, the presence of several compounds with recognized antioxidant and protective potential such as gallic acid, catechin, caffeic acid, quercetin among others was identified in *A. microcarpum* extracts (Barbosa Filho et al., 2014[6]). 

Paraquat (1,1′-dimethyl-4-4′-bipyridynium dichloride), is a quaternary ammonium herbicide widely used in agriculture. When administered *in vivo*, it undergoes NADPH-dependent reduction, generating a stable paraquat radical, which reacts with oxygen to generate superoxide anion, a reactive oxygen species (ROS) (Bus and Gibson, 1984[10]). ROS generated directly or indirectly by paraquat can cause lipid peroxidation, protein carbonylation, oxidation of protein thiols and DNA damage, leading to cell death. Substantial evidence from the literature indicates that paraquat causes oxidative stress and mitochondrial dysfunction thereby disturbing ATP production (Mitra et al., 2011[24]; Hosamani and Muralidhara, 2013[18]). Chronic paraquat exposure has been identified as a risk factor for Parkinson's disease. Numerous studies reveal that this herbicide induces dopaminergic cell loss in animal models (Liou et al., 1996[23], Franco et al., 2010[16]; Mitra et al., 2011[24]). 

The fruit fly *Drosophila melanogaster* is extensively used as an alternative model organism in biomedical research since it raises few ethical concerns and offers many benefits. Of particular importance, 75 % of the genes that cause disease in human are also found in *D. melanogaster* (Pandey and Nichols, 2011[28]; Narayanan and Rothenfluh, 2016[25]). For instance, neurodegeneration and inflammatory events with similar features to those observed in patients with Parkinson's disease have been observed in this model (Dalui and Bhattacharyya, 2014[12]). In addition, its short reproductive cycle (8-14 days) associated with the fact that it is inexpensive to maintain in the laboratory makes it an attractive model. Furthermore, this model has been shown to be a powerful model system for the study of fundamental cellular pathways responsible for metal and other chemicals induced toxicity (Ahamed et al., 2010[3]; Bonilla-Ramirez et al., 2011[8]; Abolaji et al., 2014[1]; Paula et al., 2014[29]).

Taking into account the promising potential of *A. microcarpum *as a natural antioxidant, we hypothesized that it may offer protection against paraquat toxicity. Therefore, the objective of this study was to investigate the potential toxicity and protective effect of *A. microcarpum* against locomotor deficits and oxidative stress promoted by paraquat using *D. melanogaster* as a model organism*.*

## Materials and Methods

### Material

Dimetilsulfoxide, quercetin, 5,5′-Dithiobis(2-nitrobenzoic acid), Acetylcholine iodide, 1-chloro-2,4-dinitrobenzeno, 2′,7′-Dichlorofluorescein diacetate (DCHF-DA), Folin-Ciocalteu, 2,2'-azino-bis(3-ethylbenzothiazoline-6-sulfonic acid diammonium salt, sodium acetate, HEPES, minimum 99,5 % titration, 2,4,6-Tris(2-pyridyl)-5-triazine (TPTZ), methyl viologen dichloride hydrate (Paraquat), 3-(4,5-dimethylthiazol-2-yl)-2,5 diphenyltetrazolium bromide (MTT), resazurin sodium salt, and albumin from bovine serum were purchased from Sigma-Aldrich (São Paulo, SP, Brasil). Potassium ferrocyanidetrihydrate, zinc acetate dihydrate, iron (II) ammonium sulfate hexahydrate, gallic acid monohydrate, aluminum chloride hexahydrate, potassium persulfate were purchased from Vetec Química Fina LTDA (Rio de Janeiro, RJ, BRA). All other reagents were of analytical grade. 

### Plant collection and extractions

The stem barks of *A. microcarpum* were collected from Barrero Grande, Crato-Ceará (7°22_S; 39°28_W; 892 m sea level), Brazil, in November 2011. The plant material was identified by Dr. Maria Arlene Pessoa da Silva of the herbarium Caririense Dárdano de Andrade - Lima (HCDAL) of the Regional University of Cariri (URCA) and a voucher specimen was deposited (number 6702). The fresh barks of *A. microcarpum* were macerated with 99.9 % of ethanol and water (1:1, v/v) for 3 days. The suspension was filtered and the solvent evaporated under reduced pressure, and lyophilized to obtain 490 g of crude ethanolic extract. One hundred and fifty grams (150 g) of this was partitioned with ethyl acetate and methanol to obtain 12.5 g of ethyl acetate fraction and 105.23 g of methanolic fraction (Barbosa Filho et al., 2014[6]). All the fractions were stored in the freezer and resuspended in water prior to experiments.

### Analysis of in vitro antioxidant activity of extract and fractions

The analysis of *in vitro *antioxidant potential of ethanolic extract and fractions (methanolic and ethyl acetate) were performed in 96 well plates using the EnSpire^®^ multimode plate reader (Perkin Elmer, USA) at concentrations of 1 μg/mL, 10 μg/mL, 40 μg/mL and 400 μg/mL dissolved in distilled water. 

### Total phenolics

Phenolic compounds from ethanolic extract and fractions samples were detected by the Folin-Ciocalteu method (Singleton et al., 1999[34]) with minor modifications. Briefly, 4 μL of the samples was mixed with 35 μL of 1 N Folin-Ciocalteu's reagent. After 3 min, 70 µL of 15 % Na_2_CO_3_ solution was added to the mixture and adjusted to 284 μL with distilled water. The reaction was kept in the dark for 2 h, after which the absorbance was read at 760 nm. Gallic acid was used as standard (10 - 300 μg/mL). The results were expressed as mg of Gallic acid equivalents (GAEs) per 100 g of extract or fractions.

### ABTS radical scavenging assay

The antioxidant activity of extract and fractions sample in the reaction with ABTS radical was determined according to method of Re et al. (1999[31]) with some modifications. Briefly, ABTS radical solution was generated by oxidation of solutions composed of 1 mL of 7 mM 2,2′-azino-bis(3-ethylbenzothiazoline-6-sulfonic acid) diammonium salt stock solution and 17.5 μL of 140 mM potassium persulfate (K_2_S_2_O_8_). The mixture was left to stand in the dark at room temperature for 12-16 hours before use. For the evaluation of antioxidant capacity, the ABTS solution was diluted with distilled water to obtain the absorbance of 0.700 ± 0.020 at 734 nm. 200 μL of ABTS solution were mixed with 2 μL of sample and 48 μL of distillated water in a microplate and the decrease in the absorbance was measured after 10 min. Ascorbic acid (1 mM) was used as a positive control. The radical scavenging activity (RSA) was calculated by the formula % RSA = [(AB - AA)/AB] x 100; where RSA = ABTS scavenging; AB = absorption of a blank sample (only ABTS); AA = absorption of a tested sample.

### Drosophila stock and culture

*D. melanogaster* (Harwich strain) was obtained from the National Species Stock Center, Bowling Green, OH. The flies were reared in 2.5 X 6.5 cm^2^ glass bottles containing 10 mL of standard medium mixture of 39 % coarse and 32 % medium corn flour, 10 % wheat germ, 14 % sugar, 2 % milk powder, 1 % salt, 1 % soybean flour, 1 % rye flour, 0.02 % of methylparaben and 0.02 % of lyophilized yeast. All experiments were performed with the same strain and both sexes were used at random with age between 1-4 days post eclosion.

### Treatment of flies

In order to evaluate the toxicity of *A. microcarpum * hydroethanolic extract (AMHE), metanolic fraction (AMMF) and acetate fraction (AMAF), 25 flies of both genders were exposed to 1 and 10 mg/mL of AMHE, AMMF or AMAF for 5 consecutive days. It should be stressed that the extract or fractions was mixed with the standard medium prior exposure. Treatments were compared with control group that received standard medium only. 

To evaluate the protective potential of AMHE, AMMF and AMAF against paraquat toxicity, flies were divided in groups of 25 flies of both genders and exposed to different treatments: 1 % sucrose (control group), 5 mM paraquat, 1 and 10 mg/mL of AMHE, AMMF and AMAF alone or in combination with 5 mM paraquat. All solutions were diluted in 1 % sucrose and a volume of 500 µL was added to a filter paper in the base of flies vials. The number of dead flies was registered for a period of 3 days (72 h). This was chosen on the basis that no mortality or behavioral alteration was noted. At the end of the treatment, behavioral and biochemical analyses were conducted. 

### Cellular viability of flies

The viability assay was performed by resazurin reduction assay. The method uses the indicator resazurin to measure the metabolic capacity of cells. Groups of 20 flies treated for 72 hours with extract or fractions mixed in culture medium were mechanically homogenized in 1 mL 20 mM Tris buffer (pH 7.0) and centrifuged at 1,600 g for 5 min at 4 °C. The supernatant was incubated in the Elisa plates with 20 mM Tris buffer (pH 7.0) and resazurin for 2 h. After this period, the fluorescence emission was read using EnsPire^® ^multimode plate reader (Perkin Elmer, Waltham, MA) at excitation wavelength of 560 nm and emission wavelength of 590 nm. 

### Activity of antioxidant enzymes

For analysis of antioxidant enzymes activity, twenty flies were homogenized in 200 μL of Tris buffer (20 mM, pH 7.0), then centrifuged at 1.000 g for 5 minutes at 4 °C. An aliquot of the supernatant was used for the determination of acetylcholinesterase (AchE) activity (Franco et al., 2009[15]; Ellman et al., 1961[14]). The remained supernatant was then centrifuged at 20.000 g for 30 minutes at 4 °C and then used to measure Calatase (CAT; EC 1.11.1.6), Glutathione-S-transferase (GST; EC 2.5.1.18) and superoxide dismutase (SOD, EC 1.15.1.1). CAT activity was assayed following the clearance of H_2_O_2_ at 240 nm in a reaction media containing 50 mM phosphate buffer (pH 7.0), 0.5 mM EDTA, 10 mM H_2_O_2_, 0.012 %TRITONX100 as described by Aebi (1984[2]). The GST activity was assayed following the procedure of Habig and Jakoby (1981[17]) using 1-chloro-2,4-dinitrobenzene (CDNB) as substrate. The assay is based on the formation of the conjugated complex of CDNB and GSH at 340 nm. The reaction mixture consisted of 100 mM phosphate buffer (pH 7.0), 1 mM EDTA, 1 mM GSH and 2.5 mM CDNB. SOD activity assay was performed as described by Kostyuk and Potapovich (1989[21]). The assay consists in the inhibition of superoxide driven oxidation of quercetin by SOD at 406 nm. The complete reaction system consisted of 25 mM phosphate buffer (pH 10), 0.25 mM EDTA, 0.8 mM TEMED and 0.05 μM quercetin. The enzyme activities were expressed in milliunits per milligram of total protein content, which was quantified following Bradford (1976[9]).

### Negative geotaxis assay

Locomotor ability was determined using the negative geotaxis assay as described by Coulom and Birman (2004[11]). For the experiment, five flies from each group were ice-anaesthetized and placed in a vertical plastic tube (15 cm length). After 30 minutes of recovery, the number of flies that reach 8 cm up the tube and the flies that remained below this mark after 6 seconds were registered. The experiment was repeated three times with intervals of one minute.

### DCF-DA oxidation assay

Groups of 20 flies were mechanically homogenized in 1 mL 20 mM Tris buffer (pH 7.0), and centrifuged at 1,600 g for 10 min, 4 °C. The supernatant was used to quantify 2′-7′-dichlorofluorescein diacetate (DCF-DA) oxidation as a general index of ROS formation as described by Perez-Severiano et al. (2004[30]). The fluorescence emission of DCF resulting from DCF-DA oxidation was monitored at regular intervals (488 nm excitation and 530 nm emission) in an EnsPire® multimode plate reader (PerkinElmer, USA). The graph expresses the fluorescence after 60 min of incubation with the fluorescent compound. 

### Statistical analysis

All the procedures were made in triplicates. Statistical analysis was performed using a one-way or two-way ANOVA followed by Tukey's *post hoc* test. The results were considered statistically significant when p < 0.05. 

## Results

### Phenol content and in vitro antioxidant activity

The *in vitro* antioxidant property of *A. microcarpum* crude extract and fractions evaluated by the ABTS assay is shown in Table 1(Tab. 1). The content of total phenols varied among samples. AMAF presented the higher phenolic content (103.3 ± 1.25 g GAE/100 g of sample) followed by AMHE (79.66 ± 1.74) and AMMF (70.33 ± 3.10). The antioxidant activity of *A. microcarpum* was tested by its ability to neutralize ABTS radical. AMAF exhibited highest ABTS radical scavenging activity (Table 1(Tab. 1)). The characterization of specific phenolic and flavonoid compounds was already identified in crude extract and fractions by our research group (Barbosa Filho et al., 2014[6]) using high performance liquid chromatography (HPLC). The extract and fractions contained caffeic acid, quercetin and isoquercetin among others, that can be at least in part, responsible for its antioxidant activity. 

### Survival of flies exposed to A. microcarpum

**Figure 1:**
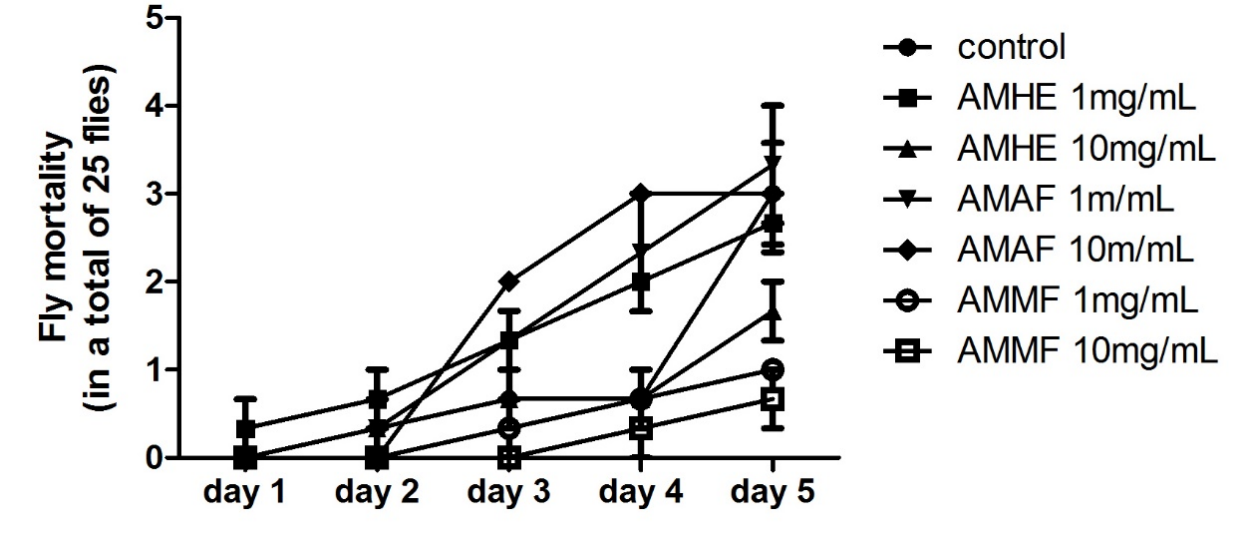
Mortality of flies exposed to *A. microcarpum. *25 flies of both sexes per group and in triplicate were exposed to *A. microcarpum* hydroalcoholic extract (AMHE), acetate fraction (AMAF) and methanolic fraction (AMMF) at different concentrations for 5 days. The number of dead flies was registered daily. No significant difference was detected.

The effects of *A. microcarpum* exposure on fly's survival were evaluated during five days. As showed in Figure 1(Fig. 1), long term consumption of extract or fractions did not affect the survival of flies when comparing to control group, even at the highest concentration tested (10 mg/mL). Further analysis was performed after 72 hours of exposure to the extract or fractions mixed with medium.

### Cell viability of flies exposed to A. microcarpum

**Figure 2:**
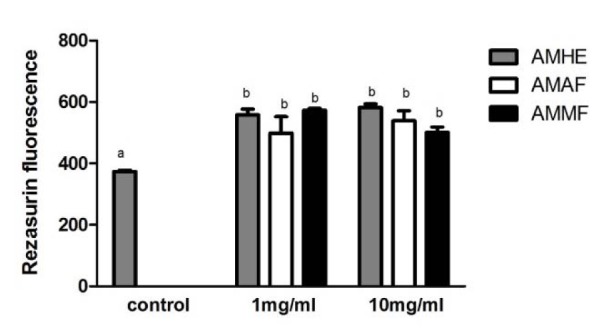
Cell viability of flies exposed to *A. microcarpum. *Flies were treated with *A. microcarpum* hydroalcoholic extract (AMHE), acetate fraction (AMAF) and methanolic fraction (AMMF) in 1 % sucrose for 72 h. After treatment, flies were homogenized, centrifuged and the supernatant was used for analysis of cellular viability through Rezaruzin reduction assay. The results are mean ± SEM of 3 independent experiments. ^a^: no difference in relation to control group;^b^: differs in relation to control group considering p< 0.05.

Flies were exposed for 72 hours to *A. microcarpum *extract or fractions. After this period, cellular viability was evaluated in the homogenates of whole body of flies through Rezaruzin reduction assay as a measurement of metabolic activity of cells. As shown in Figure 2(Fig. 2) the metabolic activity of flies was significantly increased in all concentrations of extract and fractions tested. Similar results were observed in MTT test (data not shown). 

### Antioxidant enzyme activity of flies exposed to A. microcarpum

The treatment with *A. microcarpum* caused significant increase (p < 0.05) in GST activity of flies treated with 10 mg/mL of AMAF (from 477.76 ± 92 to 770.19 ± 147.92 mU/mg of protein). Catalase activity was significantly increased in flies treated with 10 mg/mL of AMMF (from 66.77 ± 6.64 to 223.94 ± 25.92 mU/mg of protein) and AMAF (from 66.77 ± 6.64 to 220.54 ± 26.63) at 10 mg/mL. However, AMHE at all the concentrations tested did not change the activity of the enzymes when compared with the control (p > 0.05). No alteration in AchE activity was observed in response to the treatments (Table 2(Tab. 2)).

### Effects of A. microcarpum and paraquat on flies survival 

**Figure 3:**
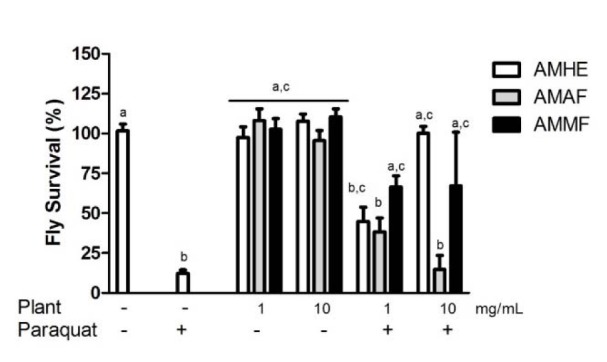
Effects of *A. microcarpum* and paraquat on flies survival. Flies were treated with the extract (AMHE) or fractions (AMAF and AMMF) alone or in combination with paraquat 5 mM for 72 days. At the end of the treatment, the number of alive flies was registered. The results are expressed as percentage of control. Each bar represents the mean ± SEM of 3 independent experiments. ^a^: no difference in relation to control group; ^b^: differs in relation to control group, ^c^: differs in relation to paraquat group considering p < 0.05.

The consumption of extract or fractions by flies did not alter survival of flies when comparing with the control (considered as 100 %). However paraquat caused a 85 % decrease in flies survivorship compared with the control (p < 0.05; Figure 3(Fig. 3)). The co-exposure of AMHE and paraquat resulted in a concentration dependent blockage of paraquat induced mortality. AMMF at concentrations of 1 and 10 mg/mL prevented against paraquat induced flies death. In contrast, AMAF did not provide protection against death caused by paraquat (Figure 3(Fig. 3)). 

### Effects of A. microcarpum and paraquat on flies locomotor activity

**Figure 4:**
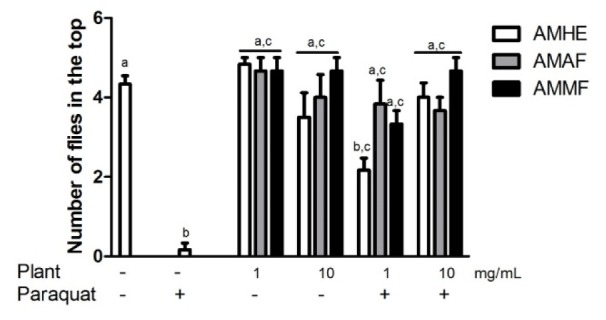
Effects of* A. microcarpum *and paraquat on flies locomotor performance. Flies were treated with the extract (AMHE) or fractions (AMAF and AMMF) alone or in combination with paraquat 5 mM for 72 days. At the end of treatment, locomotor activity was evaluated by negative geotaxis, as described in 'Materials and Methods' section. A total of 5 flies per group was used in three independent experiments. ^a^: no difference in relation to control group; ^b^: differs in relation to control group, ^c^: differs in relation to paraquat group considering p < 0.05.

Negative geotaxis consists in the ability of flies to fly vertically when startled and it is a common measure of locomotor behaviour (Rhodenizer et al., 2008[32]). Exposure to *A. microcarpum* extract and fractions did not alter locomotor performance of flies when compared with the control (p < 0.05). Exposure of flies to paraquat significantly impaired flies locomotor ability when compared with the control (Figure 4(Fig. 4), p < 0.05). About 70 % of paraquat-treated flies remained below the line, indicating locomotor deficit caused by paraquat. At concentration of 1 mg/mL, crude extract (AMHE) prevented partially while AMAF and AMMF restored the locomotor deficit caused by paraquat (Figure 4(Fig. 4)). The concentration of 10 mg/ mL of the extract and fractions completely prevented locomotor deficit caused by paraquat (Figure 4(Fig. 4)).

### Effects of A. microcarpum and paraquat on reactive species production in flies

**Figure 5:**
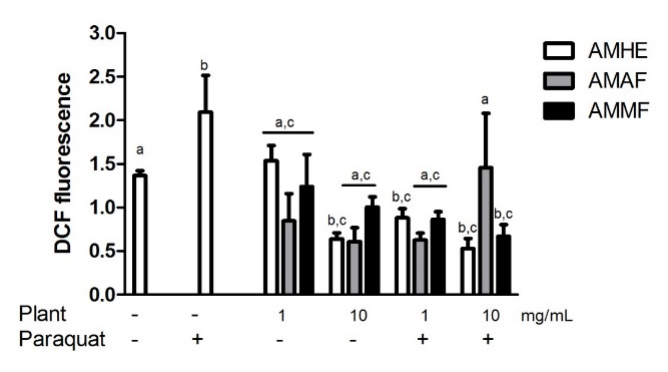
Effects of* A. microcarpum *and paraquat on ROS production. Flies were treated with the extract (AMHE) or fractions (AMAF and AMMF) alone or in combination with paraquat 5 mM for 72 days. After treatment, ROS production was measured in the supernatant of flies as an index of oxidative stress. Results are expressed as mean ± SEM of raw fluorescence as a result of DCF-DA oxidation (N=4). ^a^: no difference in relation to control group; ^b^: differs in relation to control group, ^c^: differs in relation to paraquat group considering p<0.05.

Paraquat toxicity is mainly attributed to its oxidant character causing ROS generation. As depicted in Figure 5(Fig. 5), paraquat-exposed flies showed significant increase in ROS production when compared with the control (p < 0.05). AMHE and AMAF *per se *decreased the basal level of DCF fluorescence at the highest concentration tested. Generally, AMHE and AMMF at concentration of 1 and 10 mg/mL protected flies against paraquat-induced ROS generation. AMAF showed antioxidant effect against paraquat-induced ROS production, only at 1 mg/mL (Figure 5(Fig. 5)).

## Discussion

The understanding of the etiology and prevention of neurodegenerative diseases is a challenge and so far no definite cure has been found. In this sense, there is a need to identify compounds capable of preventing and/or reversing neurological damage (Kamdem et al. 2016[20]). It is known that oxidative stress is a common factor in a variety of neurodegenerative diseases and age-related degenerative processes. Levels of reactive species, lipid oxidation products, protein oxidation and DNA oxidation are elevated in brain of Alzheimer disease patients (Franco et al., 2010[16]). In this regard, several antioxidant compounds derived from natural sources have demonstrated neuroprotective potential *in vitro* and *in vivo *models. Among these are flavonoid polyphenols like epigallocatechin 3-gallate from green tea and quercetin from apple, non-flavonoid polyphenols such as curcumin from turmeric and resveratrol from grape (Ataie et al., 2016[4]; Huang and Adachi, 2016[19]). 

A number of epidemiologic studies have found an association between Parkinson's disease and exposure to pesticides (for review: Baltazar et al., 2014[5]; Sanchez-Santed et al., 2016[33]). In this study, the neurotoxin paraquat impaired locomotor performance of flies, and decreased survivorship. These effects were not observed when *A. microcarpum* was present in the medium. This beneficial effect of *A. microcarpum* may be at least in part attributed to the presence of quercetin, isoquercetrin, ellagic acid and caffeic acid previously identified in the extract and fractions by our research group (Barbosa Filho et al., 2014[6]). These compounds are well known as potent antioxidants. 

Although the *in vitro* antioxidant potential of this plant has been demonstrated, there was no knowledge about this effect *in vivo.* Herein, the consumption of *A. microcarpum *methanolic and acetate fraction improved the endogen activity of antioxidant enzyme catalase, which is responsible for the degradation of hydrogen peroxide, a reactive species prone to cause damage to biomolecules (Aebi, 1984[2]). This effect could be attributed to a modulation of gene expression of enzyme, leading to its augmented levels. The literature shows that the consumption of cranberry extract by *D. melanogaster* induced mRNA expression of antioxidant enzymes, such as SOD and protected against damage induced by hydrogen peroxide (Wang et al., 2015[36]). Moreover fruit flies carrying copies of SOD1 and CAT genes exhibited a one-third extension of lifespan, lower amount of protein oxidative damage and delayed loss in physical performance (Orr and Sohal, 1994[26]). Thus, the induction of antioxidant enzymes activity showed in this study could be beneficial for flies and confer increased resistance to oxidative damage. 

The GST enzyme activity was augmented following treatment with acetate fraction. This enzyme participates in phase II detoxifying process and acts in the inactivation of toxic molecules from endogenous and exogenous sources converting them into water-soluble compounds. In other studies, *Ginkgo biloba* extract and metabolites was able to increase the activity of GST in *Hyphantria cunea* larvae (Pan et al., 2016[27]). In the current study, only the highest concentration of acetate fraction increased the activity of this enzyme; such fact could be attributed in part to a higher concentration of several constituents such as flavonoids and polyphenols, as verified by HPLC studies (Barbosa Filho et al., 2014[6]). High concentration of flavonoids was shown to be toxic in cell culture model causing an oxidative damage and mitochondrial failure (Lee et al., 2015[22]). Acetate fraction was not able to avoid the drop in survivorship caused by paraquat. This result draws attention to the fact that the *in vitro *antioxidant potential of the extract is not an indicative of a superior protective activity *in vivo*. Accordingly, previous study performed by our research group demonstrated a superior DPPH scavenging potential for AMAF but its protective potential in brain tissue homogenate was lower than AMHE (Barbosa Filho et al., 2014[6]).

In conclusion, our study demonstrates for the first time that hydroalcoholic extract and fractions of *A. microcarpum* are able to protect against the toxicity promoted by the neurotoxin paraquat *in vivo*. This effect could be attributed to the antioxidant potential of this plant, which was evidenced by its potential in improving activity of antioxidant enzymes and neutralizing ROS generated directly or indirectly by paraquat. Our data draw attention to the potentiality of *A. microcarpum* as a source of chemical components with neuroprotective action *in vivo *without causing toxicity. 

## Acknowledgements

The authors thank Universidade Federal do Pampa, Conselho Nacional de Desenvolvimento Científico e Tecnológico (CNPq 456207/2014-7), Fundação de Amparo à Pesquisa do Estado do Rio Grande do Sul (Fapergs 2380-2551/14-8). Dr. Jean Paul Kamdem acknowledges the financial support of FUNCAP, CNPq and CAPES.

## Conflict of interest

The authors declare that they have no conflict of interest.

## Figures and Tables

**Table 1:**
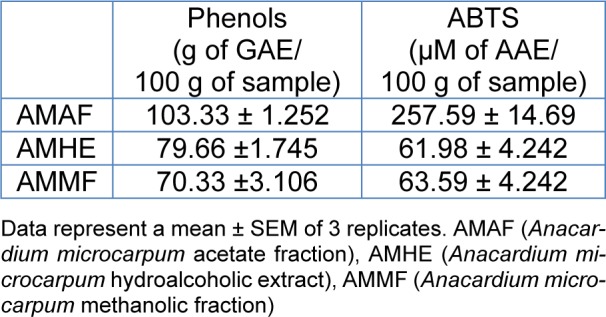
Antioxidant activity of *Anacardium microcarpum* extract and fractions

**Table 2:**
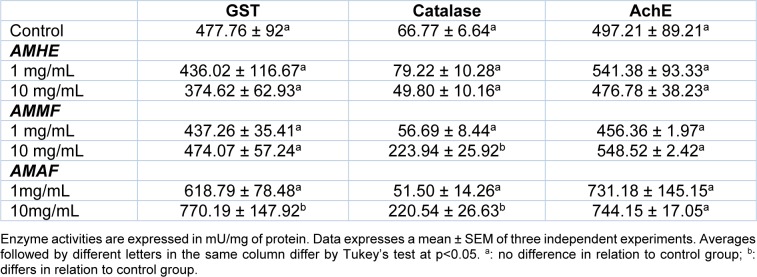
Activity of antioxidant enzymes in flies exposed to the extract or fractions of *A. microcarpum*
